# A rare presentation of central pontine myelinolysis secondary to hyperglycaemia

**DOI:** 10.1186/s12902-023-01361-y

**Published:** 2023-05-10

**Authors:** Wen-Ping Sun, Ying-Di Wang, Song Gao, Yi-Fan Wang, Da-Wei Li

**Affiliations:** 1 Department of General Practice, Songjiang Hospital Affiliated Shanghai Jiao Tong University School of Medicine, Shanghai, 201600 China; 2 Department of Urinary Surgery, Tumor Hospital of Jilin Province, Changchun, 132000 China; 3 Department of Anesthesiology, Tumor Hospital of Jilin Province, Changchun, 132000 China; 4 Department of Neurology, Shenzhen Sami Medical Center, 1 JinNiu Xi Steet, Shenzhen, 518000 P.R. China

**Keywords:** Central pontine myelinolysis, Hyperglycaemia, Osmotic demyelination syndrome

## Abstract

**Background:**

Central pontine myelinolysis (CPM) is a rare demyelinating disorder caused by the loss of myelin in the center of the basis pontis. CPM typically occurs with rapid correction of severe chronic hyponatremia and subsequent disturbances in serum osmolality. Although hyperglycaemia is recognized as a pathogenetic factor in serum osmolality fluctuations, CPM is rarely seen in the context of diabetes.

**Case presentation:**

A 66-year-old Chinese male presented with a history of gait imbalance, mild slurred speech and dysphagia for two weeks. MRI showed the mass lesions in the brainstem, and laboratory examinations showed high blood glucose and HbA1c, as well as increased serum osmolality. The patient was diagnosed with CPM secondary to hyperosmolar hyperglyceamia and received insulin treatment as well as supportive therapy. After six weeks of followup, the patient had fully recovered to a normal state.

**Conclusion:**

CPM is a potentially fatal neurological condition and can occur in uncontrolled diabetes mellitus. Early diagnosis and timely treatment are crucial for improving the prognosis.

## Background

Central pontine myelinolysis (CPM) is a demyelinating disorder caused by the loss of myelin in the centre of the basis pontis; this disorder presents clinically with quadriparesis, dysarthria, ophthalmoplegia, ataxia, psychosis, seizures, and altered mental status [1–6]. CPM was initially described in the patients with malnutrition or alcoholism and typically occurred after rapid correction of severe and chronic hyponatremia [7]. Other pathogenetic conditions, including prolonged diuretic use, burns and liver transplantation, have also been recognized as risk factors for the developmemt of CPM [8, 9]. While hyperglycaemia is a potential cause of the disturbance in serum osmolality, CPM rarely occurs secondary to the management of diabetes mellitus, especially in uncontrolled diabetes mellitus. Although there is no effective treatment for CPM, recent results suggest that the early diagnosis and improved intensive care treatment are closely associated with improved outcomes [10]. In the present case report, the patient developed CPM following uncontrolled hyperglycaemia in the absence of sodium abnormalities; he responded well to glucose control, recovering completely.

## Case presentation

A 66-year-old Chinese male presented with a two-week history of gait imbalance, mild slurred speech and dysphagia. One week previously, he had been admitted to the local hospital, and investigations showed a normal brain computed tomography (CT) and a significant increase in serum blood glucose (22.6 mmol/L). The patient was diagnosed with 2 diabetes mellitus and managed with an insulin regimen and aspirin. Due to noncompliance with the drug therapy regimen, the patient’s symptoms progressively aggravated over the ensuing week. He did not have alcohol use disorder, and there no history of any significant chronic illness or surgery. Physical examination revealed that the patient was conscious and oriented to time, place and person, and his cranial nerves, motor strength and deep tendon reflexes were normal. He had dysarthria, mild dysphagia, and ataxia with bilateral dysmetria.

**Fig. 1:**
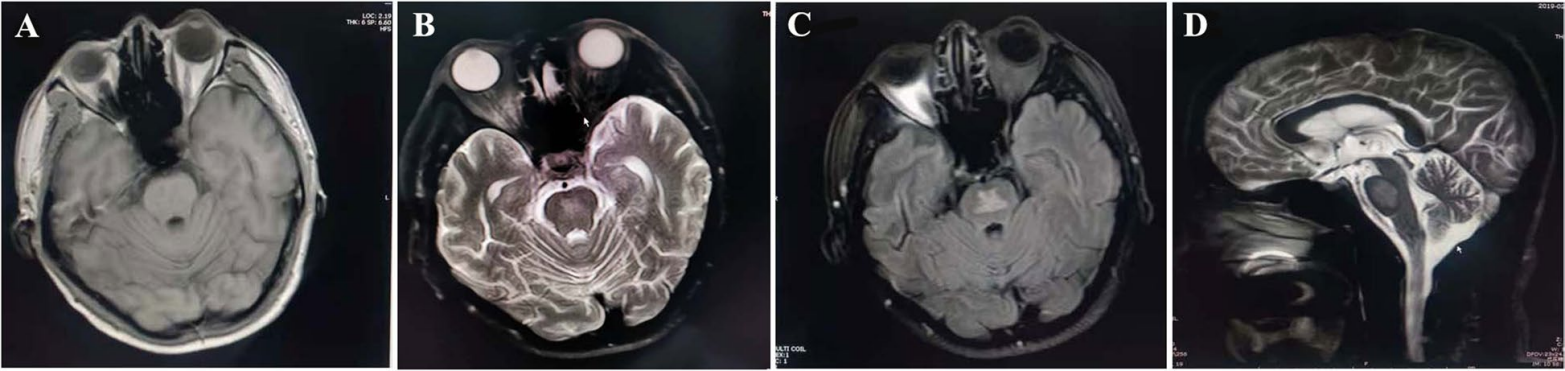
MR images of the brain. **A** T1-weighted imaging showing a mildly hypointense lesion in the central pons; (**B**, **D**) T2 and (**C**) FLAIR-weight imaging demonstrating a hyperintense lesion in the same location

Laboratory evaluation revealed the following values: random blood sugar 27.59 mmol/L, haemoglobin A1c 15%, sodium137 mmol/L, potassium 4.37 mmol/L, urea 8.49 mmol/L, and creatinine 104 µmol/L. The measured serum osmolality was 319 mmol/L. Cerebrospinal fluid analysis showed normal biochemistry and cell counts along with a negative microbiological workup. Magnetic resonance imaging (MRI) showed a sharply defined, symmetrically shaped lesion in the central pons with decreased signal intensity on T1 and increased signal intensity on T2 and fluid-attenuated inversion recovery (FLAIR)-weight imaging (Fig. 1).

A diagnosis of CPM secondary to hyperglycaemia was made, and the management of a sliding-scale subcutaneous insulin regimen was initiated. According to sliding-scale insulin therapy, the initial insulin dose was set as 12 IU. The total dose of insulin was 0.6 IU/Kg/d, where in the basal dose and the premeal bolus account for 50% of the total dose each. For three premeal bolus, the insulin dose account for 40%, 30%, and 30%, respectively. The insulin dose was adjusted dynamically according to the results of the glucose monitoring. The glucose decreased smoothly and finally attained glycemic control target. The physical rehabilitation was prescribed for pseudobulbar palsy. Gradual improvement in the patient’s gait imbalance, slurred speech, and dysphagia was noted over the ensuing week. He was discharged at the end of the second week, and no changes were evident on repeat MRI. The patient’s symptoms, however, were further recovered. After six weeks of follow-up, the patient had fully recovered to a normal state.

## Discussion

CPM is an acquired demyelinating lesion of the basis pontis associated with various clinical circumstances, including alcoholism, malnutrition, prolonged diuretic use, burns and liver transplantatipon [11–15]. The rapid correction of severe chronic hyponatremia is recognized as a common pathogenic factor triggering osmotic stress and subsequent demyelination in the central pons [16, 17]. Osmotic stress impairs the blood–brain barrier (BBB), leading to disruption of the BBB and osmotic demyelination of vulnerable cells in the brain. Pathologically, CPM is characterized by the loss and destruction of myelin sheaths, while neuronal cell bodies and axons are preserved [5]. The central pons is hightly susceptible to osmotic stress due to the rich in admixture of grey and white matter elements [11]. Demyelinating lesions can also occur at extrapontine sites including the basal ganglia, cerebellum, internal capsule and thalamus under the osmotic stress conditions, in a condition referred to as extrapontine myelinolysis (EPM); thus the syndrome consisting of CPM and EPM is termed osmotic demyelination syndrome (ODS) [16, 18]. The majority of the documented CPM cases are described secondary to disturbances in sodium homeostasis, especially in the rapid correction of severe chronic hyponatremia. However, several cases of CPM have been reported in association with the hyperosmolar hyperglycaemic state (HHS), and as a complication following by the management of HHS [19, 20]. Recently, reports have described rare cases of CPM as a complication of undiagnosed or untreated diabetes in the absence of abnormal serum sodium concentrations [2, 21]. Similar to the patients in thoses reports, our patient presented with hyperglycaemia alone and was not being treated for hyperosmolar hyperglycaemia. Hyperosmolar hyperglycaemia is associated with the changes in osmolality and the occurrence of ODS. Thus, our findings support the conclusion that the disturbance of serum osmolality is a central event in the pathogenesis of CPM and is responsible for the demyelination in the central pons, regardless of serum sodium levels.

Clinical manifestations of CPM vary based on the brain region involved and typically include spastic quadriparesis, dysarthria, dysphagia, sensory alterations, and encephalopathy of various degrees as well as ‘locked-in syndrome’in severe cases [1, 3, 5, 22]. Our patient developed a predominant clinical manifestation of ataxia along with mild dysphagia, caused by injury to corticocerebellar and corticobulbar fibres, respectively. The diagnosis of CPM depends on clinical manifestations and a characteristic radiographic appearance. The typical CPM lesion is described as a symmetric, sharply demarcated focus in the basis pontis. CT is usually normal for several days to two weeks, after which the typical hypodense lesions may be evident in the basis pontis [5]. Consistent with these reports, the brain CT of our patient was normal more than a week after the onset of symptoms. Magnetic resonance imaging (MRI) is more sensitive and reliable than CT for the diagnosis of CPM. Characteristic.

CPM lesions, located in the basis pontis, are hyperintense on T2-weighted imaging, and hypointense on T1-weighted imaging [23]. There is no effective treatment available for CPM, and the condition is managed mainly through supportive treatment. A few reports of the administration of intravenous immunoglobulin, plasmapheresis exchange or thyrotropin-releasing hormone indicate a positive effect on recovery from CPM. Limited sample sizes, however, leave the effectiveness of these therapeutic methods uncertain [16]. In the current case report, the patient was completely recovered after his blood sugar level controlled. Based on sereral previous reports and our case, timely diagnosis and treatment seem to improve recovery from hyperglycaemia-related CPM [2, 24–26].

## Conclusion

CPM is a potential complication of uncontrolled diabetes mellitus in the absence of sodium abnormalities, and MRI examination is needed to provide early clues for the diagnosis and timely treatment of this disease. Control of blood glucose should be emphasized in patients with diabetes mellitus to prevent the development of CPM.

## Data Availability

All relevant data are included in the manuscript.

## Abbreviations

CPM: Central pontine myelinolysis
BBB: Blood–brain barrier
EPM: Extrapontine myelinolysis
ODS: Osmotic demyelination syndrome
HHS: Hyperosmolar hyperglycaemia state
